# Assessment of eye care practices and health belief model factors among adult intensive care unit nurses in public hospitals of Amhara Region, Ethiopia

**DOI:** 10.1186/s12912-024-02525-4

**Published:** 2024-11-25

**Authors:** Abebe Dilie Afenigus, Helen Asmamaw Asres

**Affiliations:** 1 Department of Nursing, College of Medicine and Health Sciences, Debre Markos University, PO Box 269, Debre Markos, Gojjam Ethiopia; 2 Department of Public Health, College of Medicine and Health Sciences, Debre Markos University, PO Box 269, Debre Markos, Gojjam Ethiopia

**Keywords:** Eye care practice, Health belief model, Intensive care unit, Nurses, Public hospitals, Ethiopia

## Abstract

**Background:**

Eye care is a vital aspect of overall patient health, especially in intensive care units (ICUs) where patients face a heightened risk of ocular complications. Approximately 60% of patients with tracheal tubes and lagophthalmos develop severe ocular surface diseases, such as corneal abrasions, conjunctivitis, and exposure keratopathy, due to insufficient eye protection and lubrication. These complications can adversely affect patient outcomes, including increased mortality rates, extended hospital stays, and reduced satisfaction with care. Despite the importance of effective eye care, practices among intensive care unit nurses can be inconsistent, often influenced by their beliefs and perceptions. The Health Belief Model (HBM) offers a framework to understand these influences, highlighting how nurses’ attitudes toward eye care are shaped by their perceptions of patient severity, susceptibility to complications, perceived benefits and barriers to care, cues to action, and self-efficacy.

**Objective:**

This study aims to assess eye care practices among adult intensive care unit nurses in public hospitals in the Amhara Region of Ethiopia and to identify factors influencing these practices based on the Health Belief Model.

**Methods:**

A facility-based cross-sectional study was conducted among 213 nurses working in adult ICUs using simple random sampling. Data were collected through a structured, self-administered questionnaire and an observation checklist utilizing Kobo Collect. The data were analyzed using SPSS. Bivariable and multivariable logistic regression models were employed to identify relationships between the constructs of the Health Belief Model and eye care practices.

**Results:**

In this study, 213 of the 222 respondents participated, resulting in a 96% response rate. Among the participants, 113 nurses (53.1%; 95% CI: 46.5–59.6) demonstrated a high likelihood of providing eye care, while 100 nurses (46.9%; 95% CI: 40.4–53.5) exhibited a lower likelihood based on constructs of the Health Belief Model. Additionally, 125 nurses (58.7%; 95% CI: 52.1–65.3) had inadequate eye care practices, while 133 (62.4%; 95% CI: 55.4–69) possessed adequate knowledge about eye care. Furthermore, 113 participants (53.1%; 95% CI: 46–60.1) held a favorable attitude toward eye care. The multivariable analysis identified several factors associated with eye care practices: monthly salary (AOR = 2.4, 95% CI: 1.1–5.7), educational level (AOR = 0.2, 95% CI: 0.06–0.8), knowledge of eye care (AOR = 2, 95% CI: 1.1–3.4), and availability of eye care equipment (AOR = 0.3, 95% CI: 0.1–0.5).

**Conclusion and recommendation:**

The study reveals that fewer than half of the nurses working in adult intensive care units in public hospitals in the Amhara region provide adequate eye care, despite a strong intention to do so. Key factors influencing eye care practices include monthly salary, knowledge level, educational qualifications, and the availability of necessary equipment. To improve eye care delivery, it is essential for relevant authorities to implement targeted training and educational initiatives for nurses, thereby enhancing their skills and knowledge in eye care practices.

## Introduction

In normal conditions, the eyes are protected from injury and dryness through mechanisms like eyelid closure and the blinking reflex. These actions maintain a tear film rich in protective enzymes (like lysozyme and lactoferrin) and proteins such as immunoglobulin A, which help prevent bacterial infection [1, 2]. Additionally, blinking and tear production facilitates oxygen delivery to the cornea and clear metabolic waste via the nasolacrimal drainage system [3–6].

However, these protective mechanisms can be significantly compromised in intubated patients in intensive care unit (ICU) [7]. Factors contributing to this deterioration include altered consciousness, metabolic disturbances, impaired immunity, mechanical ventilation, use of muscle relaxants and sedatives, prolonged prone positioning, systemic diseases, paralysis, and exposure to respiratory pathogens from open suctioning [8–11]. Increased positive end-expiratory pressure (PEEP) can further exacerbate water retention and scleral edema, impairing eyelid closure and blink reflexes, which can lead to serious ocular conditions such as corneal abrasions, exposure keratopathy, and microbial infections [12, 13].

Approximately 60% of patients with tracheal tubes and lagophthalmos are at risk of severe ocular surface diseases, necessitating regular eye care such as eye cleaning, eyelid closure with lubricants, or use of moisture chambers [14–16]. In cases where passive eyelid closure is insufficient, mechanical taping methods should be considered to safeguard against ocular epithelial dryness and reduce infection risks. Additionally, special precautions are critical during suctioning to prevent the exposure of the eyes to tracheal or oropharyngeal secretions [17–19].

Despite the necessity of these routine eye care interventions, nurses often face challenges such as time constraints, limited resources, and awareness issues that hinder their ability to deliver care effectively [20, 21]. Although nurses' perception of ocular care for critically ill patients is favorable, their overall knowledge and awareness require significant improvement to meet the standards expected in an intensive care environment [22]. A study found that only 6% of ICU nurses demonstrated a satisfactory level of eye care practice [23]. The Health Belief Model (HBM) offers a valuable framework for identifying the factors that affect these practices [24].

## Theoretical framework of Health Belief Model (HBM)

**Fig. 1:**
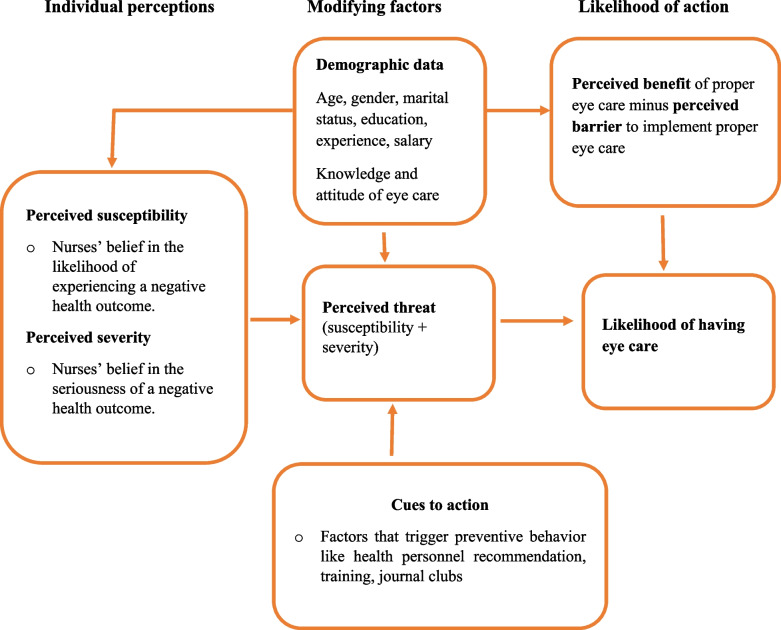
Modified Health Belief Model constructs for nurses to provide eye care in Amhara region public hospitals, 2024

The health belief model (HBM) is a widely used theoretical framework that guides interventions aimed at improving health behaviors among nurses. This model emphasizes the importance of understanding the beliefs and perceptions that influence behavior change [25, 26]. HBM comprises several core constructs, including perceived susceptibility, perceived severity, perceived benefits, perceived barriers, cues to action, and self-efficacy. These constructs are particularly relevant in the context of eye care practices for critically ill patients in ICUs (Fig. 1).

Perceived threat encompasses nurses’ subjective assessments of the seriousness and likelihood of eye problems, incorporating two key components: perceived susceptibility and perceived severity. Perceived susceptibility refers to the belief in the likelihood of experiencing a negative health outcome. In this context, it reflects nurses' beliefs about the risk that critically ill patients may develop eye issues, such as corneal abrasions or infections. Nurses often recognize that, without preventive measures, these patients face significant risks due to their medical conditions or prolonged ICU stays [26–28].

Perceived severity pertains to the belief in the seriousness of a health outcome. The extent to which nurses believe that eye health problems can lead to serious consequences (dry eyes, corneal abrasion) for their patients. Acknowledging the serious consequences can enhance nurses' commitment to implementing preventive care strategies [24, 26, 28].

Perceived benefits refer to the belief in the effectiveness of preventive behaviors. This construct emphasizes the perceived advantages of engaging in proactive eye care practices. Nurses must believe that regular assessments and protective measures will lead to improved patient outcomes, such as reduced discomfort and quicker recovery [24, 26, 28].

Perceived barrier refers to the obstacles that nurses face in implementing eye care practices, such as lack of resources, time constraints, or insufficient training. Identifying and addressing these barriers is crucial for enhancing eye care delivery [24, 28, 29].

Cues to action are factors that trigger preventive behaviors. These cues serve as stimuli that prompt nurses to engage in proactive eye care practices, which may include educational interventions, reminders, or institutional policies. Effective cues can remind nurses to prioritize eye care and enhance adherence to best practices [24, 26, 28].

Lastly, self-efficacy describes nurse’s confidence in their ability provide appropriate eye care to patients. Strengthening nurses' self-efficacy through training and support ensures they feel capable and confident in delivering effective eye care interventions [28, 30, 31].

Knowledge, attitudes, perceived susceptibility, perceived severity, perceived benefits, perceived barriers, cues to action, and demographic characteristics (including age, sex, and income) are factors in influencing individuals' behavioral changes [24, 32]. In the context of ICUs, nurses often face significant challenges in providing eye care due to the urgency of situations and the high volume of treatments required for inpatients [9]. This study aims to assess eye care practices of adult ICU nurses in public hospitals in the Amhara Region of Ethiopia and to identify factors influencing these practices, using Health Belief Model as a guiding framework.

### Objectives

- To assess eye care practices among adult intensive care unit nurses in public hospitals in the Amhara Region of Ethiopia, 2024.
- To identify the factors influencing the practices of adult intensive care unit nurses in public hospitals in the Amhara Region of Ethiopia, based on the constructs of the Health Belief Model, 2024.

### Research question

How do sociodemographic characteristics, knowledge, attitudes, and perceived barriers, as framed by the Health Belief Model, influence eye care practices among adult intensive care unit nurses in public hospitals of the Amhara Region, Ethiopia?

## Methods and materials

### Study design, study setting and period

A cross-sectional study was conducted from January 1 to March 30, 2024, among nurses working in adult intensive care units of Amhara region public hospitals with complementary approaches to the Health Belief Model. There are a total of 10 hospitals with adult ICU units, each having an average of 42 ICU nurses, which totals 420 ICU nurses. These adult ICU units serve both medical and surgical patients; however, they are not categorized into separate medical or surgical units. This information is included in the study setting of the methods section.

### Study population and eligibility criteria

All selected nurses working in adult intensive care units of Amhara region public hospitals who were available during the data collection period were the study populations whereas those nurses who were seriously ill during the data collection period were excluded from the study**.**

### Sample size and sampling procedure

The sample size was calculated using the Rao soft program by considering the following assumptions, the margin of error of 5%, 95% confidence level, the total number of nurses who work in Amhara region intensive care units (420), and 50% response distribution. Besides this, by adding a 10% non-response rate the final sample size was 222. Moreover, the participants were selected by using a simple random sampling technique.

### Variables of the study

The dependent variable is the eye care practice in the ICU whereas the independent variables were sociodemographic characteristics(age, sex, work experience, level of qualification, training, availability of eye care equipment, and monthly salary); knowledge of nurses towards eye care (risk factors of ocular surface diseases, assessment of eye and routine activities of eye care); attitude of nurses towards eye care in the ICU (nurses views on eye care priority, willingness to provide eye care, and effect of pre and post procedure handwashing, standard endotracheal suctioning, and staff education) and ICU nurses individual perceptions on patients perceived threat (perceived susceptibility, perceived severity), perceived benefit, perceived barrier and cues to action.

### Operational definitions

- *Eye care practice*: Practice is assessed by a total of 23 items that comprise components to assess hand hygiene and personal protective equipment (3 items), physical examination of patient eye (6 items), and pertinent eye care conducted for patients (14 items) by using an observation checklist of nurses practice of eye care in the ICU. Then each practice item was computed and scored as “1” for completely done items and “0” for wrongly done or missed practice. Finally, the total practice score is categorized as good practice if they have properly done ≥ 75% using the observation checklist, whereas if the practice score of eye care performed by nurses using the observation checklist is < 75%, it is considered inadequate practice [33].
- *Knowledge of eye care*: Nurses' knowledge of eye care was assessed using 14 items. Each knowledge-related item is coded “1” if nurses correctly answer it and “0” if nurses do not answer or incorrectly answer each knowledge-related item. Then those nurses who obtained 80% or more were categorized as having good eye care knowledge, whereas those who scored below 80% were categorized as having inadequate knowledge [33].
- *Attitude towards eye care*: Those nurses who scored the mean score or above of attitude-related items were categorized as having a favorable attitude, whereas those nurses who scored below the mean score were categorized as having an unfavorable attitude.
- *Perceived susceptibility* is assessed by three Likert scale items ranging from strongly disagree (1 score) to strongly agree (5 score). Those participants who had the mean or above score of susceptibility-related items are categorized as having high perceived susceptibility, whereas those who scored below the mean are considered to have low perceived susceptibility.
- *Perceived seriousness/severity* is assessed by three Likert scale items ranging from strongly disagree (1 score) to strongly agree (5 score). Those participants who had the mean or above score of perceived severity-related items are categorized as having high perceived severity, whereas those who scored below the mean are considered to have low perceived severity.
- *Likelihood of eye care*: It is measured by using the perceived benefits and perceived barriers of the HBM. If the sum score of perceived benefit minus the sum score of perceived barriers is positive (≥ 1), there is a high likelihood or intention of practicing eye care, whereas if the sum score of perceived benefit minus the sum score of perceived barriers is negative (< 1), there is a low likelihood of practicing eye care.

### Data collection procedure and tool

The study employed structured self-administered questionnaires and observation checklists to collect data on ICU nurses' practices regarding eye care for critically ill adult patients, utilizing constructs from the Health Belief Model. The questionnaire used in this study was developed after an extensive review of relevant literatures and existing clinical guidelines in the field [23, 34, 35]. The total time taken to complete the entire interview and observation could range from 30 to 55 min. The data collectors gave the study participants a brief overview that explained the study's goals and the significance of their participation. Then, a survey was conducted to volunteer intensive care nurses. Moreover, 10 diploma nurses collected the data and 3 BSc nurses supervised the process.

### Data quality assurance

Before the commencement of actual data collection, the principal investigator provided two days of training for data collectors and supervisors regarding the purpose of the study, the questionnaire or tool, how to respect respondent rights and privacy, and the methods of data collection and supervision. The questionnaire was carefully designed, and the English version was used for data collection. Before the actual data collection time, the questionnaire (tool) was pretested on 5% of the total sample (*n* = 12) at Finote Selam General Hospital. Then, based on the findings of the pretest, the questions were modified and improved for wording and structure. Similarly, the items were arranged based on the content validity index (CVI) format and sent to two intensivists via email to give their evaluation of clarity and relevance. Based on their responses, item content validity index (I-CVI) scores will be calculated by dividing the expert agreement by the number of experts, and finally, the average of I-CVI scores across all items will be computed. Based on this procedure, the overall score content validity index (S-CVI) of the items was 0.88, which indicates the tool is acceptable. Moreover, the reliability of the tool was assessed by Cronbach alpha, with overall alpha coefficients of 0.8. Similarly, the data obtained from each questionnaire was examined and verified for accuracy, completeness, and consistency daily.

### Data processing and analysis

The data were checked for completeness, cleaned, coded, and entered into Kobo collect software, and then analyzed using SPSS (version 27). Bivariate and multivariable logistic regression models were used. Crude Odds Ratios (COR) with 95% confidence intervals were estimated in the bivariable logistic regression analysis to assess the association between each independent variable and outcome variable. In the bivariable logistic regression, variables with *P*-value < 0.2 were fitted into the multivariable logistic regression analysis. Finally, Adjusted Odds Ratios (AOR) with their 95% confidence intervals (CI) were estimated to assess the strength of association and variables with *P*-value < 0.05 were considered statistically significant factors. Model fitness was assessed by Hosmer and Lemeshow’s goodness of fit test and multicollinearity between each independent variable was checked and no correlation was detected between two independent variables.

## Results

### Sociodemographic characteristics

The research involved 222 nurses, with 213 willingly consenting and 9 declining or not filling out questionnaires, resulting in a response rate of 96%.

Out of 213 respondents, 146 (68.5%) nurses received training regarding eye care in the ICU, and their mean age is 28.9 years with SD of 5.4 years. Besides this, the majority, 156 (73.2%) of the participants, were bachelor's degree holders. In addition, more than half, 111 (52.1%) were females, and 104 (48.8%) had available guidelines in their working units (Table 1).

**Table 1:** Sociodemographic characteristics of nurses working in adult intensive care units in Amhara region, 2024 (*n* = 213)

Variable	Frequency (%)
**Sex**	
Male	102(47.9)
Female	111(52.1)
**Age**	
22- 32 years	181(85)
≥ 33 years	32(15)
**Marital status**	
Married	122(57.3)
Single	71(33.3)
Divorce	20(9.4)
**Educational level**	
Diploma	28(13.1)
Bachelor degree	156(73.2)
Master’s degree	29(13.6)
**Eye care training**	
Yes	146(68.5)
No	67(31.5)
**Role of nurse**	
Head nurse	15(7)
Staff nurse	198(93)
**Monthly salary**	
< 6000.00 ETB^a^	64(30)
6000.00—8000.00 ETB	96(45.1)
≥ 8000.00 ETB	53(24.9)
**Equipment availability to give eye care?**	
Yes	112(52.6)
No	101(47.4)
**Availability of eye care guideline**	
Yes	104(48.8)
No	109(51.2)
**Experience in adult ICU**	
1–5 years	101(47.4)
6–10 years	89 (41.8)
≥ 11 years	23(10.8)

^a^ *ETB* Ethiopian birr

#### Eye care practice

The practice of eye care includes a total of 23 items and 3 major subthemes, such as hand hygiene and personal protective equipment (PPE), physical examination of patient eyes, and pertinent eye care provided for patients based on observation checklists. Among the total participants, 88 (41.3%; 95% CI: 34.7–47.9) had good eye care practice, whereas 125 (58.7%; 95% CI: 52.1–65.3) had inadequate practice. Besides this, 204 (95.8%) of the participants washed their hands before the procedure, 150 (70.4%) assessed the patient's blink reflex, 161 (75.6%) inspected eye movement and gaze, and 144 (67.6%) took special precautions for their eyes while suctioning to prevent exposure. Similarly, 135 (63.4%) grade the severity of lagophthalmos; 130 (61.03%) intervene based on the grade of exposure; and 138 (64.8%) clean patients’ eyes with saline-soaked gauze or close their eyelids for patients with or at risk of lagophthalmos (Table 2).

**Table 2:** Description of correct (yes) and incorrect (no) practice recommendations for eye care among nurses working in Amhara region adult ICU using observation checklist, 2024

No	Practice related items checklist	Yes (%)	No (%)
	**Hand hygiene and PPE**		
1.	Wash hand before procedure	204(95.8)	9(4.2)
2.	Wash hand after procedure	192(90.1)	21(9.9)
3.	Wear gloves and other PPE as necessary	156(73.2)	57(26.8)
	**Physical examination**		
4.	Assess signs of eye infection and inflammation	130(61)	83(39)
5.	Inspect eye for drainage, lesion, and irritation	177(83.1)	36(16.9)
6.	Perform pupillary examination	176(82.6)	37(17.4)
7.	Assess blink reflex	150(70.4)	63(29.6)
8.	Assess and ensure patients eyes are closed on daily basis	119(55.9)	94(44.1)
9.	Inspect eye movement and note symmetry	161(75.6)	52(24.4)
	**Eye care**		
10.	Explain procedure to patient	171(80.3)	42(19.7)
11.	Apply clean moistened gauze with normal saline or sterile water and gently wipe each eye from inner to outer canthus of eye	108(50.7)	105(49.3)
12.	Use eye tape in those with incomplete lid closure	106(49.8)	107(50.2)
13.	Providing eye care for patients with mechanical ventilation	154(72.3)	59(27.7)
14.	Take special precautions to eye while suctioning to prevent aerosol exposure	144(67.6)	69(32.4)
15.	Performing suctioning while standing beside, not above, the patient’s bed and covering patient’s eyes	163(76.5)	50(23.5)
16.	The frequency of eye cleansing should vary with the frequency of eye intervention required	174(81.7)	39(18.3)
17.	Administrating appropriate eye lubricants, drop or ointment	105(49.3)	108(50.7)
18.	Ensuring patients eyes are not exposed to aspirates during tracheal or oropharyngeal suction procedures	164(77)	49(23)
19.	Ensuring that the endotracheal or the tracheostomy tube is correctly fixed in place (on a daily basis)	162(76.1)	51(23.9)
20.	Patients with or at risk of Lagophthalmos should clean their eyes with saline-soaked gauze, close their eyelids with ocular lubricant, or create a moisture chamber with polyethylene wrap	138(64.8)	75(35.2)
21.	Do you grade severity of lagophthalmos as grade 0 = lids completely closed; grade 1 = any conjunctival exposure; and grade 2 = any corneal exposure	135(63.4)	78(36.6)
22.	Does your action based on grading of exposure like grade 0 exposure (i.e., no exposure) require no action; grade 1 exposure requires lubrication; grade 2 exposure needs lubrication and taping of the lids with micropore tape along the lash margin	130(61.03)	83(38.97)
23.	Treat eye infection with broad spectrum antibiotics until result of swab available or ophthalmic consultation for eye problems	146(68.5)	67(31.5)

### Likelihood of eye care (Constructs of Health Belief Model)

In this study, we assessed ICU nurses’ perceptions regarding the susceptibility and severity of eye care issues for adult ICU patients within the framework of the Health Belief Model (HBM). According to the operational definitions, 142 (66.7%) nurses exhibited high perceived susceptibility to eye problems in ICU patients, while 71 (33.3%) nurses reported low perceived susceptibility. Additionally, 147 (69%) participants acknowledged high perceived severity associated with potential eye complications, whereas 66 (31%) perceived low severity.

The analysis of perceived benefits and barriers provided critical insights into the likelihood of nurses engaging in eye care practices. A positive balance, where the sum score of perceived benefits exceeded the sum score of perceived barriers was observed in 113 (53.1%; 95% CI: 46.5–59.6) nurses, indicating a strong likelihood of participation in eye care activities. Conversely, 100 (46.9%; 95% CI: 40.4–53.5) nurses displayed a negative balance, suggesting that significant barriers outweighed the perceived benefits, which may impede their commitment to prioritizing eye care interventions.

Furthermore, an overwhelming majority, 209 (98.1%) participants, strongly agreed that ICU patients are at risk for developing eye problems. Many nurses identified key barriers to effective eye care provision, including lack of time, complexity of care, staffing shortages, and associated costs. These findings emphasize the urgent need to address these perceived barriers to improve eye care delivery within intensive care settings (Table 3).

**Table 3:** Constructs of health belief model among nurses working in Amhara region public hospitals adult ICU, 2024

No	Statements	Strongly disagree (%)	Disagree (%)	Neutral (%)	Agree(%)	Strongly agree (%)
	**Perceived susceptibility**					
1.	Patients in ICU are at risk of getting eye infection	-	7(3.3)	10(4.7)	37(17.4)	159(74.6)
2.	It is possible that patients in ICU get eye problems	-	-	6(2.8)	16(7.5)	191(89.7)
3.	It is likely that patients in ICU get eye problems	-	-	-	4(1.9)	209(98.1)
	**Perceived severity**					
4.	Eye problems/infections in ICU patients has serious negative consequences	5(2.3)	2(0.9)	-	25(11.7)	181(85)
5.	Eye problems are extremely harmful	9(4.2)	5(2.3)	5(2.3)	2(0.9)	192(90.1)
6.	Getting eye problems in the ICU is a sure death sentence	27(12.7)	15(7.0)	4(1.9)	3(1.4)	164(77)
	**Perceived benefit**					
7.	Eye care is effective in preventing eye infections/problems	-	-	-	31(14.6)	182(85.4)
8.	Providing eye care prevent eye problems	-	-	-	35(16.4)	178(83.6)
9.	If I give consistent eye care, patients less likely get eye problems	-	-	-	21(9.9)	192(90.1)
10.	Providing eye care to treat eye problems will reduce vision loss	10(4.7)	7(3.3)	3(1.4)	49(23)	144(67.6)
	**Perceived efficacy**					
11.	I am able to provide eye care	-	-	-	4(1.9)	209(98.1)
12.	Providing eye care is easy for me	3(1.4)	-	-	9(4.2)	201(94.4)
13.	I provide ophthalmic consultation for eye problems	-	10(4.7)	10(4.7)	13(6.1)	180(84.5)
	**Perceived barrier**					
14.	Providing eye care is time consuming	24(11.3)	27(12.7)	10(4.7)	21(9.9)	131(61.5)
15	There is shortage of staff to provide eye care	8(3.8)	11(5.2)	5(2.3)	12(5.6)	177(83.1)
16.	Providing eye care is complex (too much documentation)	11(5.2)	15(7)	7(3.3)	22(10.3)	158(74.2)
17.	Providing eye care will cost a lot	-	11(5.2)	8(3.8)	8(3.8)	186(87.3)

### Cues to action

**Fig. 2:**
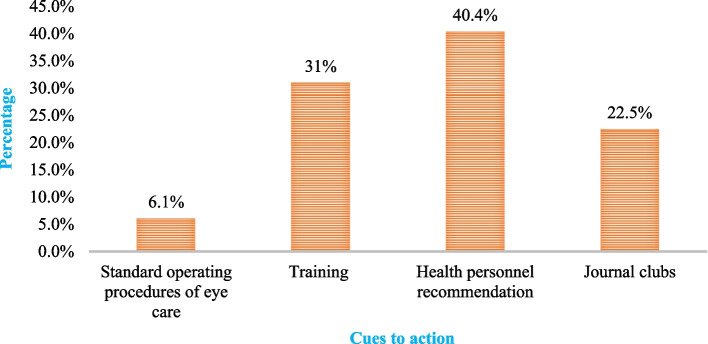
The cues to action that influence adult ICU nurses’ eye care practices in Amhara region public hospitals, 2024

In this study, different sources of information influenced the eye care practices of nurses within the framework of the HBM. The findings revealed that 13(6.1%) participants relied on standard operating procedures for eye care information, indicating a low awareness of established protocols. In contrast, a significant proportion of participants- 66 (31%) reported receiving training, which may enhance their perceived self-efficacy and readiness to provide quality eye care. Notably, 86(40.4%) participants cited recommendations from health personnel as a crucial source of information, suggesting that social influence and professional guidance play a vital role in shaping their practices. Additionally, 48(22.5%) participants obtained information through journal clubs, highlighting the importance of continuous education and peer discussions in reinforcing eye care knowledge (Fig. 2).

### Knowledge of nurses towards eye care

Fourteen items that aimed to assess the knowledge of nurses about eye care were provided to nurses. These items comprise risk factors for ocular surface diseases, eye assessment, and facts on routine activities of eye care (Table 4). Based on the operational definition stated in the methods section, 133 (62.4%; 95% CI: 55.4–69) were knowledgeable, whereas the remaining 80 (37.5%) of the respondents had inadequate knowledge of eye care.

**Table 4:** Description of correct(true) and incorrect(false) responses of nurses working in adult ICU in Amhara region, 2024 (*n* = 213)

No	Knowledge related items	True (%)	False (%)
1.	All patients should receive regular eye cleaning to remove debris, secretions, dried ointment and / or other ocular medications	194(91.1)	19(8.9)
2.	ICU patients are high risk of xerosis and exposure keratopathy	193(90.6)	20(9.4)
3.	Sedatives or positive pressure ventilation disturbs blink reflex	152(71.4)	61(28.6)
4.	Lagophthalmos is a potential risk factor for eye disorders	164(77)	49(23)
5.	Eyelid closure is important criterion in assessing eye disorders in ICU	139(65.3)	74(34.7)
6.	Pull lower lid down and instill ointment onto eye between lower lid and conjunctiva if ointment is applied	166(77.9)	47(22.1)
7.	Positive pressure ventilation aggravates chemosis	189(88.7)	24(11.3)
6.	Assessment of eyelid closure must be done at the onset of the care plan, and then regularly throughout the patient’s stay	150(70.4)	63(29.6)
9.	With moisten swab, gently clean along the eyelashes in one movement, from inner to outer canthus	178(83.6)	35(16.4)
10.	Covering patients’ eyes or performing suctioning not above patients bed helps prevent splash of secretions to patients’ eye	198(93)	15(7)
11.	Appropriate size for eye pad and cover is necessary	204(95.8)	9(4.2)
12.	Lubricants and lid taping are used for ventilated patients with absent blink reflex and corneal exposure	202(94.8)	11(5.2)
13	The right direction for applying adhesive tape on eyelids for closing the eyes are holding the tape horizontally	196(92)	17(8)
14.	Ocular lubrication or protection of corneas with polyethylene chamber is most effective intervention to prevent corneal abrasion	196(92)	17(8)

**Fig. 3:**
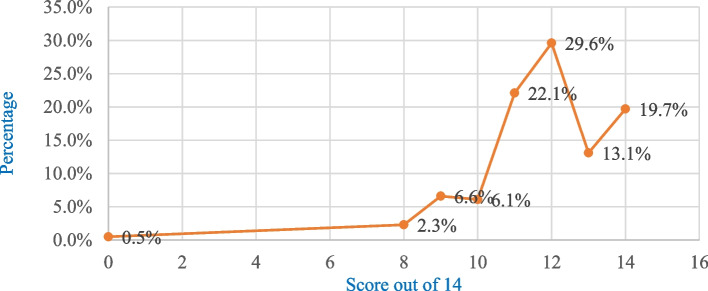
Knowledge score of adult ICU nurses from 14 items, Amhara region public hospitals, 2024

Out of 14 items that aimed to assess ICU nurses’ knowledge, 1 (0.5%) scored 0, 5 (2.3%) scored 8, 14 (6.6%) scored 9, 13 (6.1%) scored 10, 47 (22.1%) scored 11, 63 (29.6%) scored 12, 28 (13.1%) scored 13, and 42 (19.7%) scored 14 (Fig. 3).

### Attitude of nurses towards eye care

Moreover, a total of seven items were used to assess the attitude of nurses towards eye care in the ICU. The items comprise nurses’ views on the priority of eye care in the ICU, willingness to provide eye care, the effects of pre-and post-procedure handwashing, standard endotracheal suctioning, and staff education (Table 5). Based on the operational definition stated in the methods section, 113 (53.1%; 95%CI: 46–60.1) of the participants had a favorable attitude, while 100 (46.9%; 95%CI: 39.9–54) had an unfavorable attitude toward eye care.

**Table 5:** Attitude of nurses working in adult intensive care units in Amhara region towards eye care, 2024

No	Attitude related questions	Strongly disagree (%)	Disagree (%)	Neutral (%)	Agree (%)	Strongly agree (%)
1.	Do you think priority is given to eye care for patients in ICU	11(5.2)	13(6.1)	12(5.6)	16(7.5)	161(75.6)
2.	Do you think pre and post procedure hand washing prevent or reduce eye disorders	15(7)	10(4.7)	6(2.8)	17(8)	165(77.5)
3.	Do you think eye care is important for patients receiving mechanical ventilation	-	-	-	35(16.4)	178(83.6)
4.	Are you willing to provide eye care for patients with mechanical ventilation	-	-	5(2.3)	21(9.9)	187(87.8)
5.	Staff education on eye care has effect on preventing eye disorders	10(4.7)	11(5.2)	7(3.3)	5(2.3)	180(84.5)
6.	Eye care has effect on preventing eye disorders	-	-	-	46(21.6)	167(78.4)
7.	Standard endotracheal suctioning has effect on reducing incidence of eye disorders	16(7.5)	10(4.7)	13(6.1)	11(5.2)	163(76.5)

### Factors affecting eye care practice in adult ICU

In bivariable logistic regression analysis, variables with a *P*-value < 0.2 were sex, monthly salary, qualification, knowledge level, attitude, perceived severity, eye care equipment availability, and perceived susceptibility. However, in multivariable analysis, only four variables were identified as significantly associated with factors of eye care (Table 6). Nurses earning a monthly salary between 6000.00 and 8000.00 ETB exhibited a 2.4-fold increase in the odds of engaging in eye care practices compared to their counterparts earning 6000.00 ETB (AOR = 2.4, 95% CI: 1.1–5.7). Additionally, those with adequate knowledge demonstrated double the odds of performing eye care compared to nurses with inadequate knowledge (AOR = 2, 95% CI: 1.1–3.4). Regarding nursing qualifications, diploma nurses were significantly less likely to perform eye care, with an 80% reduction in odds compared to master's degree holders (AOR = 0.2, 95% CI: 0.06–0.8). Furthermore, the availability of eye care equipment was a crucial factor; nurses working in intensive care units without access to such equipment had 70% lower odds of performing eye care than those with the necessary resources (AOR = 0.3, 95% CI: 0.1–0.5). These findings underscore the significant impact of salary, knowledge, qualifications, and equipment availability on the practice of eye care among ICU nurses.

**Table 6:** Bivariable and multivariable logistic regression analysis of associated factors of eye care practice at Amhara region public hospitals, 2024

Variables	Eye care		COR (95% CI)	AOR (95% CI)	*P*- value
	**Yes (%)**	**No (%)**			
**Sex**					
Male	41(46.6)	61(48.8)	1	1	
Female	47(53.4)	64(51.2)	1.1(0.6, 1.8)	1.6(0.8, 3.2)	0.2
**Monthly salary**					
< 6000.00 ETB	20(22.7)	44(35.2)	1	1	
6000.00—8000.00 ETB	43(48.9)	53(42.4)	1.9(1.1, 4.1)	2.4(1.1, 5.7)	**0.04**
≥ 8000.00 ETB	25(28.4)	28(22.4)	1.1(0.5, 2.1)	0.6(0.2, 1.6)	0.4
**Knowledge to eye care**					
Adequate knowledge	48(54.5)	85(68)	1.7(1.1, 3.1)	2(1.1, 3.4)	**0.04**
Inadequate knowledge	40(45.5)	40(32)	1	1	
**Perceived severity**					
High	60(68.2)	87(69.6)	1	1	0.9
Low	28(31.8)	38(30.4)	1.1(0.5, 1.9)	0.9(0.5, 1.5)	
**Educational level**					
Diploma	13(14.8)	15(12)	0.3(0.1, 1.1)	0.2(0.06, 0.8)	**0.02**
Bachelor	68(77.3)	88(70.4)	0.4(0.2, 1.1)	0.2(0.08, 0.6)	0.05
Masters	7(8)	22(17.6)	1	1	
**Attitude**					
Favorable	49(55.7)	64(51.2)	1	1	
Unfavorable	39(44.3)	61(48.8)	0.8(0.4, 1.4)	0.9(0.4, 1.6	0.7
**Eye care equipment availability**					
Yes	58(65.9)	54(43.2)	1	1	
No	30(34.1)	71(56.8)	0.3(0.2, 0.6)	0.3(0.1, 0.5)	** < 0.001**
**Perceived susceptibility**					
High	58(65.9)	84(67.2)	1	1	
Low	30(34.1)	41(32.8)	1.1(0.5, 1.8)	0.9(0.5, 1.9)	0.9

## Discussion

The findings of this study emphasize the importance of the Health Belief Model (HBM) in understanding the factors influencing nurses' intentions and likelihood to practice eye care in the intensive care unit (ICU). This study showed that a majority of nurses perceived high susceptibility (66.7%) and high severity (69%) regarding eye problems in ICU patients, which are critical constructs of the HBM. These perceptions align with previous research indicating that healthcare professionals who recognize the severity of a health issue are more likely to engage in preventive practices [27, 28].

The analysis of perceived benefits versus barriers further illuminated the factors influencing nurses' engagement in eye care. Over half of the participants (53.1%) showed a positive balance where perceived benefits outweighed barriers, suggesting a strong motivation to participate in eye care activities. Conversely, nearly half (46.9%) perceived barriers that hindered their commitment to these interventions. This finding highlights the complexity of healthcare decision-making, where perceived barriers such as time constraints, complexity of care, and staffing shortages can significantly impact the quality of care delivered [36, 37].

The study revealed that 41.3% of participants exhibited good(adequate) eye care practices, a finding that contrasts with a previous study in West Bank hospitals, where only 25.7% had good practices [38]. This indicates significant regional differences in eye care adherence, emphasizing the necessity for localized interventions to standardize and improve eye care protocols.

Knowledge about eye care was notably high, with 62.4% of nurses demonstrating adequate knowledge regarding eye care, compared to only 47% in a similar Saudi Arabian study [22]. This discrepancy highlights the potential for targeted education initiatives to further decrease preventable eye complications and enhance patient care outcomes in ICU settings.

Furthermore, the analysis of attitudes toward eye care showed that 53.1% of participants held a favorable view(attitude), which is considerably more positive than findings from Beni-Suef University Hospital in Egypt, where only 8% of nurses had a favorable attitude [23]. However, it contrasts with an Iranian study, where all participating nurses expressed a positive attitude [39]. These variations emphasize the need for ongoing education and awareness campaigns to address the minority of nurses with unfavorable attitudes, thereby ensuring comprehensive adherence to eye care protocols.

Importantly, the financial aspects of nursing practice were also revealed in this study. Nurses earning a monthly salary between 6000.00 and 8000.00 ETB were 2.4 times more likely to perform eye care compared to those earning less than 6000.00 ETB. This aligns with the previous research [40] indicating that financial incentives can significantly influence nurses' engagement and empowerment in patient care, ultimately leading to improved outcomes.

Moreover, nurses with adequate knowledge were twice as likely to perform eye care as those with inadequate knowledge. This finding is consistent with studies from Jordan [41], Iran [42], Egypt [43], and Turkey [44], reinforcing the correlation between knowledge and practice. This relationship suggests that enhancing nurses' knowledge through structured training and education can lead to better-informed decisions and practices in eye care.

Furthermore, diploma nurses were 80% less likely to perform eye care compared to their master's degree counterparts. This is consistent with a review study [45]. This finding calls attention to the importance of advanced education and training in improving nursing practices such as eye care, particularly in specialized areas like ICUs.

Lastly, nurses working in intensive care units lacking adequate eye care equipment were 70% less likely to perform eye care compared to those with access to such resources. This finding is supported by different studies and standards [4, 46]. This finding emphasizes the critical role that appropriate equipment for effective eye care practices. It highlights the urgent need for healthcare facilities to prioritize the procurement and maintenance of essential eye care equipment.

### Strength and limitation of the study

The major strength of the study is assessing the factors of eye care using the health belief model. In addition, a major limitation of the study is that the pre-test, conducted on only 5% of the total sample, may be small to adequately assess the internal consistency of the questionnaire. Besides this, HBM fails to address social factors that influence nurses' behavior in providing eye care.

## Conclusion

Less than half of the nurses in adult intensive care units at public hospitals in the Amhara region are providing adequate eye care. Key factors associated with effective eye care include monthly salary, knowledge of eye care, educational level, and the availability of necessary equipment.

## Recommendations

To address the inadequate provision of eye care among nurses in adult intensive care units at public hospitals in the Amhara region, relevant authorities should implement the following recommendations. First, increasing the monthly salaries of nurses could incentivize greater engagement in eye care practices, as fair compensation enhances job satisfaction and encourages nurses to prioritize patient care, including eye care interventions. Additionally, developing ongoing training programs is essential to improve nurses' knowledge of eye care; this could include workshops, seminars, and online courses focusing on the latest best practices and procedures, as well as the importance of eye care in the ICU setting. Emphasizing practical skills and hands-on training will empower nurses to apply their knowledge effectively. Furthermore, it is crucial to ensure the availability of essential eye care equipment in all intensive care units; establishing a standardized inventory of necessary tools will facilitate better eye care practices and enable nurses to perform interventions with confidence. Finally, future research should concentrate on identifying and evaluating specific interventions aimed at addressing the barriers influencing eye care practices.

## Data Availability

Data is provided within the manuscript or supplementary information files.
